# Human Neutrophil Response to *Pseudomonas* Bacteriophage PAK_P1, a Therapeutic Candidate

**DOI:** 10.3390/v15081726

**Published:** 2023-08-12

**Authors:** Dwayne R. Roach, Benoît Noël, Sylvie Chollet-Martin, Mathieu de Jode, Vanessa Granger, Laurent Debarbieux, Luc de Chaisemartin

**Affiliations:** 1 Institut Pasteur, Université Paris Cité, CNRS UMR6047, 75015 Paris, France; dwayne.roach@sdsu.edu (D.R.R.); mathieu.dejode@sciensano.be (M.d.J.); 2 Department of Biology, San Diego State University, San Diego, CA 92182, USA; 3 INSERM UMR-996, Inflammation, Microbiome and Immunosurveillance, Faculté de Pharmacie, Université Paris-Saclay, 91400 Orsay, France; benoit.noel.upsud@gmail.com (B.N.); sylvie.chollet-martin@u-psud.fr (S.C.-M.); vanessa.granger@u-psud.fr (V.G.); 4 APHP, Service Auto-Immunité et Hypersensibilités, HUPNVS, Hôpital Bichat, 75018 Paris, France

**Keywords:** Phage therapy, immune response, safety, innate immunity, in vitro assays, cytokines

## Abstract

The immune system offers several mechanisms of response to harmful microbes that invade the human body. As a first line of defense, neutrophils can remove pathogens by phagocytosis, inactivate them by the release of reactive oxygen species (ROS) or immobilize them by neutrophil extracellular traps (NETs). Although recent studies have shown that bacteriophages (phages) make up a large portion of human microbiomes and are currently being explored as antibacterial therapeutics, neutrophilic responses to phages are still elusive. Here, we show that exposure of isolated human resting neutrophils to a high concentration of the *Pseudomonas* phage PAK_P1 led to a 2-fold increase in interleukin-8 (IL-8) secretion. Importantly, phage exposure did not induce neutrophil apoptosis or necrosis and did not further affect activation marker expression, oxidative burst, and NETs formation. Similarly, inflammatory stimuli-activated neutrophil effector responses were unaffected by phage exposure. Our work suggests that phages are unlikely to inadvertently cause excessive neutrophil responses that could damage tissues and worsen disease. Because IL-8 functions as a chemoattractant, directing immune cells to sites of infection and inflammation, phage-stimulated IL-8 production may modulate some host immune responses.

## 1. Introduction

As a key component of the innate immune system, neutrophils are generally considered rapid responders in the first line of defense against pathogen invasion. They are the predominant population among leukocytes and can clear pathogens by a number of mechanisms, including phagocytosis, production of reactive oxygen species (ROSs) and other antimicrobial products, as well as neutrophil extracellular traps (NETs) [1,2]. The role of neutrophils has long been considered restricted to the initial phase of defense. However, recent evidence indicates that there is functional heterogeneity and plasticity among neutrophils that shape both innate and adaptive immune responses [3,4]. To this end, neutrophils are regularly associated with inflammation and disease, while also promoting inflammation resolution and homeostasis.

The human microbiota is the aggregate of more than 100 trillion symbiotic microorganisms that live on and within the body, including bacteria, archaea, eukaryotic viruses and bacteriophages (phages) [5,6]. It is now acknowledged that the human microbiota affects host physiology to a great extent, including host immunity and homeostasis [5,7]. Recently, resident phages’—a diverse group of viruses that infect bacteria—community structure and composition have been shown to be altered during inflammatory diseases, such as inflammatory bowel disease [8,9], periodontal disease [10] and diabetes [11]. Although phages are not human pathogens, Duerkop and Hooper hypothesize that phages may trigger antiviral defenses [6] because they can elicit immune responses [12,13]. More recently, phages were associated with the efficacy of fecal microbiota transplantations [14]. This suggests that phages play an unidentified role in affecting host immunity.

Moreover, to address the rise of multidrug-resistant infections, phage therapy, the use of phages as antibacterials, is supported by an increasing number of successful case reports in Europe and USA [15,16,17]. The mechanism by which phages exert their therapeutic action has generally been considered to be via their capacity to lyse bacterial cells. However, studies have shown that phages and the innate immune system work synergistically to eliminate bacterial infections [18,19]. In particular, neutrophils were necessary for the successful phage therapy of acute pneumonia in mice [19]. This study was performed with the *Pseudomonas aeruginosa* phage PAK_P1, which has been thoroughly investigated by our team as an efficient candidate for treating experimental pulmonary infection in mice [19,20,21]. This phage is a double-stranded DNA (93 kb) myovirus with an 80 nm diameter capsid and 130 nm long tail that displays a strictly lytic lifecycle [20]. It belongs to the *Pakpunavirus* genus that includes, so far, only phages infecting *P. aeruginosa* [22]. The synergistic action of phage PAK_P1 with the immune response and the involvement of neutrophils in the efficacy of phage therapy call for possible phage–neutrophil interactions, which remain poorly studied [19].

Here, we co-incubated the *P. aeruginosa* phage PAK_P1 with human peripheral blood neutrophils, resting and ex-vivo activated, and measured a large panel of neutrophil responses. Using increasing neutrophil:phage ratios up to 1:10,000, we found no evidence supporting the fact that phage PAK_P1 affects phagocytosis, oxidative burst, or NETosis. The only significant signal that was detected was a moderate increase of IL-8 at the highest neutrophil:phage ratio.

## 2. Materials and Methods

### 2.1. Bacteria Culture and Phage Purification

*P. aeruginosa* strain PAK [23] was cultured in Luria Broth (LB; BD Biosciences, Rungis France) medium at 37 °C, 130 rpm and normoxic atmosphere. Phage PAK_P1 was enriched by cultivation in exponentially growing PAK cells after inoculation at a virus-to-bacterium ratio of 1:10. A series of purification steps were performed to reproducibly generate pure, clean and concentrated phage stocks: (1) phage lysate was centrifuged at 8000× *g* for 30 min; (2) tangential-flow ultrafiltration, diafiltration in TN buffer (10 mM Tris, 150 mM NaCl, pH 7.5) and concentration; (3) cesium chloride step density gradient ultracentrifugation followed by 24 h PBS dialysis; (4) endotoxin removal by 3 passages through an Endotrap Spin Column (Lionex GmbH, Braunschweig, Germany), as per the manufacturers’ protocol; and (5) 0.22 µm syringe filter sterilization followed by 4 °C until use [19]. The endotoxin level was quantified (0.5 ng mL^−1^ of phage stocks) with the EndoZyme II Recombinant Factor C kit (Biomérieux, Marcy l’étoile, France), according to the manufacturer’s protocol. Purified phage stocks (4 × 10^11^ plaque-forming unit mL^−1^) and dilutions in Hank’s balanced saline solution (HBSS Gibco^TM^, Thermo Fischer Scientific, Waltham, MA, USA) were titrated on lawns of strain PAK.

### 2.2. Neutrophil Isolation and Simulation

Neutrophils were separated from human peripheral blood collected from healthy donors on EDTA by negative magnetic sorting (MACSxpress, Miltenyi Biotec, Bergisch Gladbach, Germany), according to the manufacturer’s instructions. Neutrophils were washed once in HBSS and resuspended at 3 × 10^6^ mL^−1^ in HBSS, and purity >98% and viability >99% was confirmed by flow cytometry, as previously reported [24].

### 2.3. In Vitro Exposure of Neutrophils with Phage PAK_P1 and/or Physiological Agonists

Equal volumes (50 µL) of phage solution or buffer control and neutrophils were mixed to give a final neutrophil concentration of 1.5 × 10^6^ mL^−1^, which was chosen according to standard conditions previously reported [25,26]. Inflammatory responses were triggered as previously described [24,27]: either 25 nM phorbol myristate acetate (PMA, protein kinase C activator), 5 µM calcium ionophore (A23187), 20 µM platelet-activating factor (PAF, activates G protein-coupled receptors), 100 µg mL^−1^ Zymosan A (Dectin-1 agonist), 5 µg mL^−1^ *Staphylococcus aureus* peptidoglycan (PGN, TLR2 agonist), or 100 µg mL^−1^ ovalbumin/anti-ovalbumin immune complex at a 1:5 ratio in PBS (FcγRs agonist), all added at the same time as phages, respectively. All stimuli were from Sigma-Aldrich (Saint Louis, MI, USA).

### 2.4. Quantification of Cell Surface Activation Markers Expression

After incubation at 37 °C in a standard cell culture incubator (5% CO_2_, H_2_0), cells were incubated with fluorochrome-conjugated monoclonal antibodies at 4 °C for 30 min in darkness using standard procedures [26]. The following antibodies were tested: APC-Cy7 anti-CD66b, PE-Cy7 anti-CXCR1, APC anti-CD11b (Miltenyi Biotec), FITC anti-CXCR2, PE anti-CD62L and APC anti-HLA-DR (BD Biosciences) [28]. The mean fluorescence intensity (MFI) was acquired using an Attune Nxt cytometer (Thermo Fischer Scientific) with at least 400,000 events per sample.

### 2.5. Phagocytosis Quantification

Neutrophil phagocytosis was assayed by measuring after 90 min incubation in darkness with increasing concentrations of pHrodo Red Zymosan BioParticles (Thermo Fischer Scientific) conjugates at 5–50 µg mL^−1^, as per the supplier’s instructions and as already reported [26]. Phagocytosis was monitored by an increase in particle fluorescence in neutrophil acidic endosomal compartments using flow cytometry.

### 2.6. Luminol-Amplified ROS Chemiluminescence Assay

The production of reactive oxygen species (ROSs) was evaluated by chemiluminescence, as previously described [24]. Briefly, neutrophils (1.0 × 10^6^ mL^−1^) were suspended in HBSS in the presence of luminol (100 μM) for 10 min at 37 °C. Cells were then stimulated with phages (cell:phage ratio from 1:10 to 1:10,000), with or without Zymosan A (5 µg mL^−1^) or PMA (25 nM). Chemiluminescence was evaluated with a luminometer (Tristar LB941, Berthold Technologies, Thoiry, France), where light emission was recorded in relative luminescence units (RLUs) during 40 min at 37 °C. The area under the curve (AUC) was calculated for each sample tested in triplicate.

### 2.7. Neutrophil Extracellular Traps Release

Extracellular DNA release was measured by fluorescence, as described previously [26]. Briefly, 1.0 × 10^6^ mL^−1^ neutrophils were co-incubated in a cell-culture incubator at 37 °C, 5% CO_2_, H_2_0, with HBSS (negative control), phage PAK_P1 alone (cell:phage ratio from 1:10 to 1:10,000), or PAK_P1 with either PMA, A23187, PAF, or IC for 3 h in the presence of 5 µM Sytox Green (Thermo Fischer Scientific). Samples were analyzed by a TristarTM LB941 microplate reader (Berthold Technologies). Fluorescence was monitored every 15 min, and the fluorescence of the unstimulated sample was subtracted as background from each sample tested in triplicate.

### 2.8. Interleukin-8 (IL-8) Production

Neutrophils were co-incubated with HBSS (negative control), phage PAK_P1 alone (cell:phage ratio from 1:10 to 1:10,000), or PAK_P1 with either PAF or PGN at 37 °C, 5% CO_2_, for 18 h. Cell-free supernatant was collected, and IL-8 levels were measured by sandwich ELISA (hIL-8 Quantikine kit, Bio-techne, Minneapolis, MN, USA), according to the manufacturers’ instructions, with a Multiskan EX spectrophotometer (Thermo Fischer Scientific).

### 2.9. Cell Death

To determine the percentage of cells actively undergoing apoptosis (apoptotic rate), neutrophils (1.0 × 10^6^.mL^−1^) were co-incubated with phages at multiple cell:phage ratios (1:10 to 1:10,000) at 37 °C, 5% CO_2_ for 3 h or 18 h and 5 µL of FITC Annexin V (FITC Annexin V Apoptosis Detection Kit I, BD Biosciences), according to the manufacturer’s instructions. Staining with Annexin V was used in conjunction with the vital dye 7-amino-actinomycin (7-AAD) to show membrane permeability by flow cytometric analysis. In addition, cell viability was evaluated using Trypan blue staining (Sigma-Aldrich).

### 2.10. Statistical Analyses

A nonparametric Mann–Whitney U-test and Kruskal–Wallis test were used to evaluate differences among the groups. Statistical analyses were performed using Prism 8 (GraphPad, San Diego, CA, USA). Results are shown as the mean ± SEM, and *p* < 0.05 was considered statistically significant.

## 3. Results

### 3.1. Neutrophil Response to Phages

**Figure 1:**
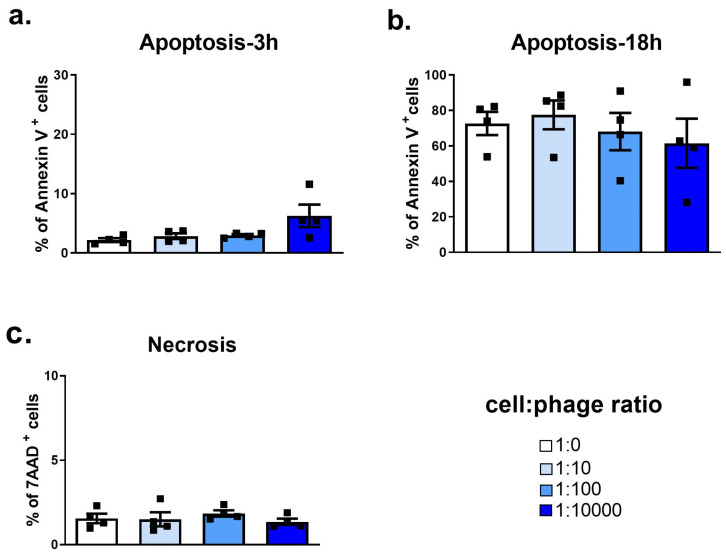
Neutrophil cell death in response to phage PAK_P1. Isolated human peripheral neutrophils were co-incubated with increasing amounts of purified phage PAK_P1. (**a**,**b**) Percent of annexin-V-positive neutrophils indicative of cell apoptosis after (**a**) 3 h and (**b**) 18 h co-incubation. (**c**) Percent of 7-AAD-positive neutrophils indicative of necrosis after 18 h of co-incubation.

**Figure 2:**
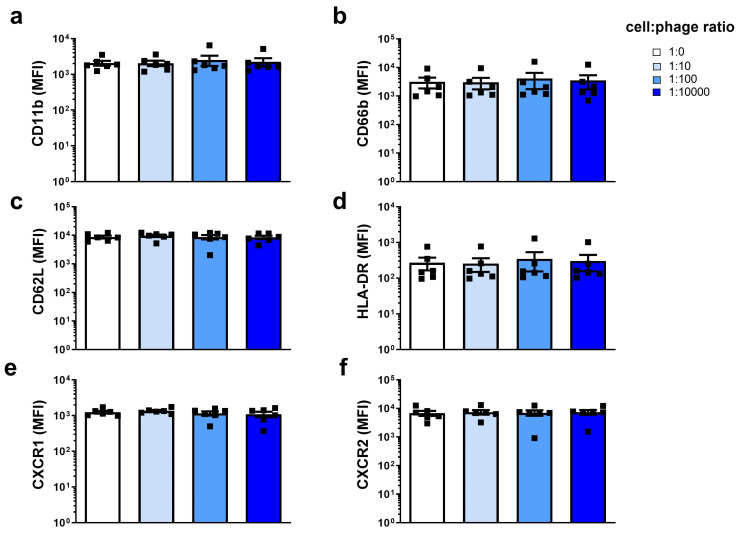
Neutrophil surface marker expression in response to phage PAK_P1. Following 18 h of co-incubation of isolated human peripheral neutrophils with an increasing amount of purified phage PAK_P1, fluorochrome-conjugated antibodies were added, and the mean fluorescence intensity (MFI) is indicated for (**a**) CD11b, (**b**) CD66b, (**c**) CD62L, (**d**) HLA-DR, (**e**) CXCR1, and (**f**) CXCR2 markers.

**Figure 3:**
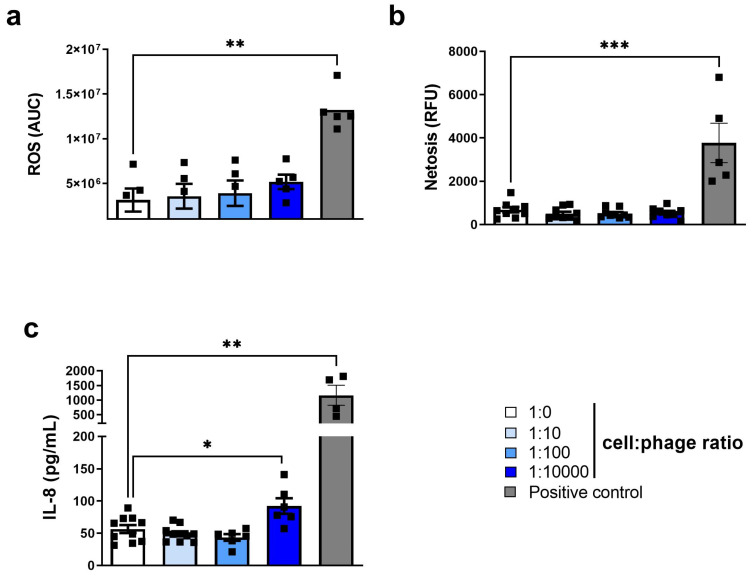
Resting neutrophil responses to phage PAK_P1. Isolated human peripheral neutrophils were co-incubated with an increasing amount of purified phage PAK_P1. (**a**) ROS production is shown as luminescence area under curve (AUC) after 40 min co-incubation. (**b**) Extracellular DNA release is shown as relative fluorescence units (RFUs) after 3 h co-incubation. (**c**) Interleukin 8 (IL-8/CXCL8) secretion is quantified after 18 h co-incubation. Positive control corresponds to PMA (25 nM) for (**a**) and (**b**) and to 5 µg mL^−1^ PGN for (**c**). Results are shown as mean + SEM, *n* = 4–9 per group; * *p* < 0.05, ** *p* < 0.01, *** *p* < 0.0001, Mann–Whitney test.

We first established the potential effects on resting neutrophils of purified phage PAK_P1 compared to the HBSS diluent control. Phage PAK_P1 was found to neither induce nor delay neutrophil apoptosis after either short (3 h) or long (18 h) co-incubations (Figure 1a,b). In addition, phages did not induce necrosis, even at the high cell:phage ratio of 1:10,000 (Figure 1c and Figure S1). We next quantified neutrophil surface activation marker expression (CD11b, CD62L, CD66b and HLA-DR) as well as chemokine receptors CXCR1 and CXCR2 (Figure 2a–f). The expression of these surface markers was not modified by phage exposure. We next assessed neutrophil antimicrobial functions in response to the phage PAK_P1. ROS production is a crucial reaction that occurs in neutrophils to degrade internalized particles and microbes. Figure 3a shows that although phages appeared to cause a slight increase in ROS production as the cell:phage ratio increased, the induction was not significant. Neutrophils have also been shown to kill pathogens by releasing web-like structures of chromatin and granules, called NETs (1). Phage PAK_P1 alone did not trigger NETs, either (Figure 3b). Although phage PAK_P1 seemed to have little effect on human neutrophils, we found that neutrophils secreted twofold higher amounts of IL-8 (CXCL8) after 18 h of co-incubation with phage PAK_P1 when at the highest 1:10,000 cell:phage ratio compared to the control (*p* < 0.01; Figure 3c). Together, these data show that phages do not modify neutrophil activation markers or trigger anti-microbial mechanisms. However, because IL-8 is involved in neutrophil activation and chemoattraction of other immune cells, human neutrophils may still sense phage virions as foreign invaders.

### 3.2. Neutrophil Responses to Inflammatory Stimuli Are Not Influenced by Phages

**Figure 4:**
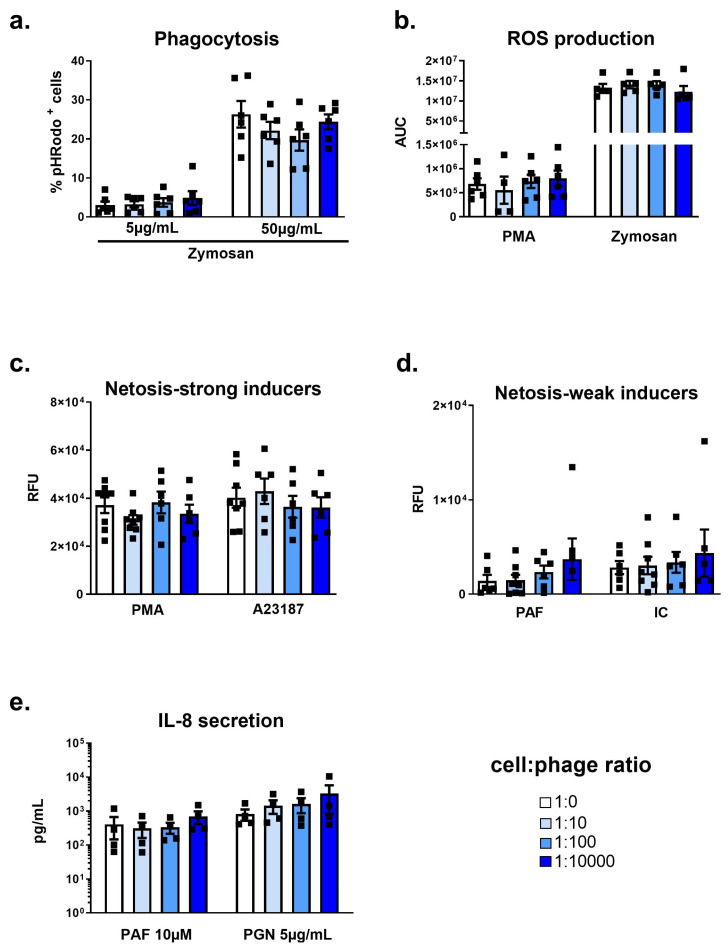
Activated human neutrophil responses to phage PAK_P1. Isolated human peripheral neutrophils were activated with either fungal glycan (Zymosan), phorbol myristate acetate (PMA), bacterial peptidoglycan (PGN), calcium ionophore (A23187), soluble immune complexes (ICs) or platelet-activation factor (PAF) and co-incubated with increasing amounts of purified phage PAK_P1. (**a**) Percent of pHRodo-positive low (5 µg mL^−1^) or high (50 µg mL^−1^) Zymosan-stimulated neutrophils after a 90 min co-incubation with phages. (**b**) Mildly (PMA) or strongly (Zymosan) activated neutrophil ROS production shown as luminescence area under curve (AUC) after 40 min co-incubation with phages. (**c**,**d**) Strongly (PMA and A23187) (**c**) or weakly (PAF and IC) (**d**) activated neutrophil extracellular DNA release shown as relative fluorescence units (RFUs) after 3 h co-incubation. (**e**) Activated neutrophil interleukin 8 (IL-8) secretion after 18 h co-incubation. Data shown as mean + SEM, *n* = 4–8 per group.

Beyond characterizing phage effects on resting blood neutrophils from healthy controls, we also questioned whether phages could potentially upregulate activation to other agonists, resulting in exacerbated inflammation. Indeed, it was suggested that certain phages induce downregulation of inflammatory responses [29]. To evaluate the potential for the modulation of activation and functional capacity, we performed additional co-incubations with phages and neutrophil physiological agonists. We found that the neutrophil phagocytic capacity of pH-Rodo Zymosan particles was not perturbed by co-incubation with phage PAK_P1 (Figure 4a). Likewise, phage PAK_P1 did not modulate oxidative burst in neutrophils stimulated with PMA (specific activator of protein kinase C) (Figure 4b, left) or the fungal TLR2 ligand Zymosan A (Figure 4b, right).

Next, we observed that phage PAK_P1 was not found to modulate activation-induced NETosis regardless of the stimulus (PMA and AA23187: strong stimuli, and PAF or IC: weak stimuli) (Figure 4c,d). Lastly, the release of IL-8 during the PAF or *S. aureus* PGN activation of neutrophils was not modulated during phage co-exposure compared to unexposed controls (Figure 4e).

Together, phage PAK_P1 did not significantly modulate the main functions of activated neutrophils with either weak or strong physiological or artificial agonists.

## 4. Discussion

In this study, we show that a high concentration of purified *P. aeruginosa* phage PAK_P1 triggered low IL-8 (CXCL8) production in freshly isolated human blood neutrophils. However, phage exposure did not further affect resting neutrophil apoptosis or induce necrosis, oxidative burst, and NET release. Activation-induced neutrophil effector responses were also unaffected by phage PAK_P1 co-exposure. Our findings contrast with those by Przerwa et al., who found that neutrophil exposure to the *Escherichia coli* phage T4 elicited weak oxidative burst, a critical antimicrobial mechanism of neutrophils [30]. Furthermore, Miedzybrodzki et al. showed that co-exposure of the phage T4 with either *E. coli* cells or *E. coli* lipopolysaccharide (LPS) dampened neutrophil ROS production [31]. However, Borysowski et al. found that exposure to the gram-positive *S. aureus* phage A3/r did not increase granule marker expression in neutrophils [32]. Previously, we showed that in mouse lung tissues, the differential production of cytokines after exposure to 10^9^ PFU of PAK_P1 did not elicit inflammatory cytokine responses [19]. However, IFNγ and TNFα productions were significantly lower in phage-exposed compared to PBS-exposed lungs [19]. It appears that phage effects on neutrophils are mild overall and that differences in neutrophil responsiveness may be phage-strain-specific.

Phage PAK_P1 at a high cell:phage ratio of 1:10,000 induced a significant twofold increase in pro-inflammatory chemokine IL-8 release [33]. IL-8 plays a role in the chemoattractant cytokine network and has a distinct target specificity for the neutrophils. In contrast, IL-8 only has weak effects on other blood cells [34]. The response of neutrophils to IL-8 is characterized by the trafficking of neutrophils across the vascular wall, the release of granule-derived enzymes, and other intra- and extracellular changes [34]. Eukaryotic viruses, such as the herpes simplex virus (HSV) and Epstein–Barr virus (EBV), have been shown to also stimulate IL-8 production. However, a major distinction is that other neutrophil effector responses are elicited, such as apoptosis and oxidative burst [35,36]. Indeed, these were not observed during phage PAK_P1 co-incubation. Globally, most human-infecting viruses are recognized by neutrophils by engaging their nucleic acid sensors, such as endosomal Toll-Like Receptor (TLR) 7, 8, and 9, and cytoplasmic Retinoic acid Inducible Gene-1 (RIG-1) and STimulator of IFN Genes (STING). In some cases, viral surface proteins can also engage a neutrophil membrane receptor, such as the Triggering Receptor Expressed on Myeloid cells (TREM-1) for Marburg or Ebola virus, or TLR4 for Respiratory Syncytial Virus [37,38,39]. Engagement of these receptors elicits a strong and global response of neutrophils. The very high dose of phage necessary to obtain a response in our setting is therefore not in favor of a strong interaction of viral proteins with surface receptors or of an active phagocytosis of phages. Primary sequences of phage proteins largely diverge from those of eukaryotic virus; however, it is possible that the three-dimensional conformations of some phage proteins display enough similarity to TLR ligands to elicit a weak response. Another possibility would be the internalization of some phage particles by micropinocytosis leading to their degradation and subsequent DNA release in endosomes, which would be then sensed by TLR9 [39].

However, a major challenge to deciphering anti-phage immune responses is the separation of phages from bacterial cell debris, such as endotoxin (i.e., LPS), peptidoglycan, exotoxins, flagella, nucleic acids and other compounds. If not adequately removed, these gross impurities could trigger inflammatory responses. Importantly, neutrophils express an abundance of bacteria-specific receptors, including the LPS-sensing TLR 4 [1]. Phage PAK_P1 preparations were determined to contain 0.5 ng mL^−1^ endotoxin. For comparison, most in vitro IL-8 secretion studies of co-incubated isolated human neutrophils had between 10 ng mL^−1^ and 1 µg mL^−1^ of endotoxin to induce expected levels of effector responses [40,41]. In addition, LPS typically enhances neutrophil activity toward subsequent viral stimuli [42], but we found no exacerbation of the neutrophil response to agonists (e.g., PMA, A23187, PAF, Zymosan A, or PGN). Therefore, it remains unclear if the weak remaining traces of endotoxins or other bacterial debris in phage preparations were in part responsible for the induction of neutrophil IL-8 secretion [43].

Although neutrophils are the first and predominant immune cell population recruited to an infected site, it remains unclear if this occurs when triggered by phage signals. Importantly, our results suggest that phages are unlikely to stimulate neutrophils into inadvertently releasing their intracellular toxic contents and causing collateral tissues damage. As a major component of the human microbiota [6], phages do not appear to play a major role in diseases where neutrophils have been implicated [44,45]. As antibacterial therapeutic agents, phages have been shown to work in concert with neutrophils to cure bacterial infection [19]. During treatment, phages do not appear to enhance neutrophil effector responses. However, since IL-8 has other biological functions besides a central role in inflammation [4], we cannot exclude that phage-stimulated IL-8 production may have a slight modulation effect on host immune responses. It should also be mentioned that these assays were not performed in the presence of actively replicating phages, as this process requires the presence of bacteria that would, by themselves, affect the neutrophil response.

The interaction between human immune cells and phages, which either make up part of the human microbiota or are administered as human therapeutics, remains underappreciated in relation to the vast genomic diversity of phages [13,46]. Our study strongly suggests that the *P. aeruginosa* phage PAK_P1 does not influence the inflammatory functions of human neutrophils. Therefore, adverse reactions to this phage used as an antibacterial agent should be limited. However, in the context of some diseases, such as cystic fibrosis, one must keep in mind that other inflammatory factors may modulate this phage-induced IL-8 release. While most phages may display similar safety characteristics, it is likely that given the diversity of phages, some could elicit stronger responses, which justifies innate immune response monitoring during phage treatments. 

## Data Availability

Raw data are available upon request to corresponding authors.

## Supplementary Materials

The following supporting information can be downloaded at: https://www.mdpi.com/article/10.3390/v15081726/s1, Figure S1: Resting human neutrophil necrosis after phage PAK_P1 co-incubation.
