# Supplementary material for: Human Neutrophil Response to Pseudomonas Bacteriophage PAK_P1, a Therapeutic Candidate
Source: Viruses. 2023 Aug 12;15(8):1726. doi: 10.3390/v15081726 (PMC10458410; doi:10.3390/v15081726)
Supplement: Supplementary file 1 [file viruses-15-01726-s001.zip › viruses-2540157-supplementary.pdf]

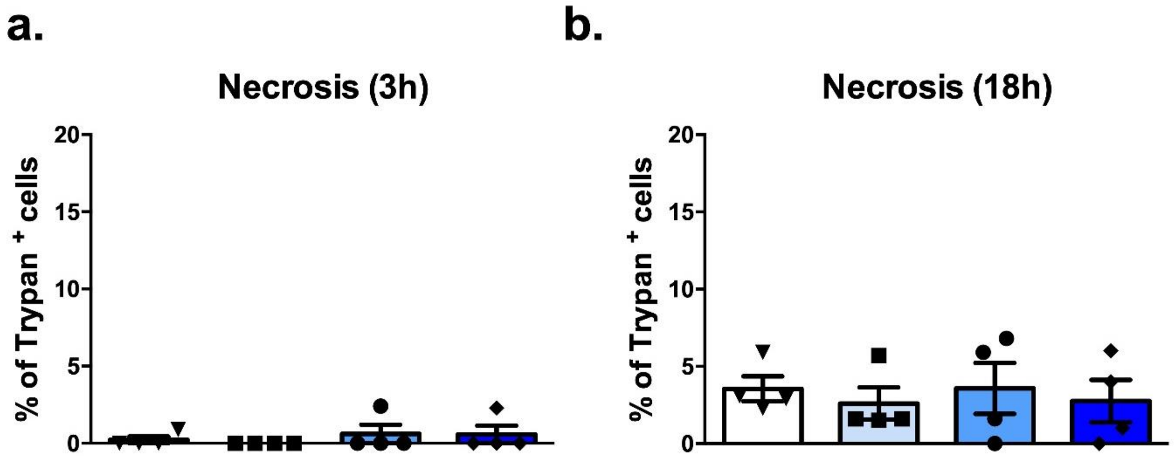

Supplementary Figure S1. Resting human neutrophil necrosis after phage PAK_P1 co-incubation. Percent of Trypan blue positive human peripheral neutrophils after (**a)** 3 h and **(b)** 18 h co-incubation with increasing amounts of purified phage PAK_P1. Data shown as mean +SEM, n= 4 per group.
